# Biomarker Evidence of Axonal Injury in Neuroasymptomatic HIV-1 Patients

**DOI:** 10.1371/journal.pone.0088591

**Published:** 2014-02-11

**Authors:** Jan Jessen Krut, Tomas Mellberg, Richard W. Price, Lars Hagberg, Dietmar Fuchs, Lars Rosengren, Staffan Nilsson, Henrik Zetterberg, Magnus Gisslén

**Affiliations:** 1 Institute of Biomedicine, Department of Infectious Diseases, University of Gothenburg, Gothenburg, Sweden; 2 Department of Neurology, University of California San Francisco, San Francisco, California, United States of America; 3 Division of Biological Chemistry, Biocenter, Innsbruck Medical University, Innsbruck, Austria; 4 Institute of Neuroscience and Physiology, Department of Neurology, the Sahlgrenska Academy at the University of Gothenburg, Gothenburg, Sweden; 5 Mathematical Sciences, Chalmers University of Technology, Gothenburg, Sweden; 6 Institute of Neuroscience and Physiology, Department of Psychiatry and Neurochemistry, the Sahlgrenska Academy at the University of Gothenburg, Gothenburg, Sweden; 7 UCL Institute of Neurology, Queen Square, London, United Kingdom; Institut National de la Santé et de la Recherche Médicale, France

## Abstract

**Background:**

Prevalence of neurocognitive impairment in HIV-1 infected patients is reported to be high. Whether this is a result of active HIV-related neurodegeneration is unclear. We examined axonal injury in HIV-1 patients by measuring the light subunit of neurofilament protein (NFL) in CSF with a novel, sensitive method.

**Methods:**

With a cross-sectional design, CSF concentrations of neurofilament protein light (NFL) (marker of neuronal injury), neopterin (intrathecal immunoactivation) and CSF/Plasma albumin ratio (blood-brain barrier integrity) were analyzed on CSF from 252 HIV-infected patients, subdivided into untreated neuroasymptomatics (n = 200), HIV-associated dementia (HAD) (n = 14) and on combinations antiretroviral treatment (cART) (n = 85), and healthy controls (n = 204). 46 HIV-infected patients were included in both treated and untreated groups, but sampled at different timepoints. Furthermore, 78 neuroasymptomatic patients were analyzed before and after treatment initiation.

**Results:**

While HAD patients had the highest NFL concentrations, elevated CSF NFL was also found in 33% of untreated neuroasymptomatic patients, mainly in those with blood CD4+ cell counts below 250 cells/μL. CSF NFL concentrations in the untreated neuroasymptomatics and treated groups were equivalent to controls 18.5 and 3.9 years older, respectively. Neopterin correlated with NFL levels in untreated groups while the albumin ratio correlated with NFL in both untreated and treated groups.

**Conclusions:**

Increased CSF NFL indicates ongoing axonal injury in many neuroasymptomatic patients. Treatment decreases NFL, but treated patients retain higher levels than controls, indicating either continued virus-related injury or an aging-like effect of HIV infection. NFL correlates with neopterin and albumin ratio, suggesting an association between axonal injury, neuroinflammation and blood-brain barrier permeability. NFL appears to be a sensitive biomarker of subclinical and clinical brain injury in HIV and warrants further assessment for broader clinical use.

## Introduction

HIV invades the central nervous system (CNS) shortly after transmission where it can then be detected in cerebrospinal fluid (CSF) throughout the course of untreated systemic infection in most patients including the majority of patients without neurological symptoms in the earlier phase of infection [1], [2]. Despite the lack of symptoms, this infection is accompanied by elevated markers of intrathecal immunoactivation, including elevated CSF white blood cell counts and neopterin concentrations [2], [3]. Without treatment, approximately 20–30% of patients develop HIV associated dementia (HAD), characterized by cognitive and motor impairment with major impact of functional capacity and lifespan [4]. With the introduction of combination antiretroviral therapy (cART), the incidence of HAD has been markedly reduced in developed countries, and even severely impaired patients have improved after treatment initiation [5]–[8]. However, despite virological suppression, long-term treated patients may complain of memory difficulties, mental slowing, attention deficits and other symptoms of neurological impairment, and a number of studies have found the prevalence of milder forms of HIV-associated neurocognitive disorders (HAND) including asymptomatic neurocognitive impairment (ANI) and HIV-associated mild neurocognitive disorder (MND) to be as high as 35–69 percent [9]–[11].

Currently, the formal diagnosis of HAND, including particularly its milder forms, relies on the results of neuropsychological testing compared to normative controls, and hence confounded by other conditions affecting performance and may be overestimated using currently recommended methods [9], [10] [12]. Importantly, these testing methods do not distinguish residual from ongoing brain injury, an issue with particular implications for antiviral or other therapies, since approaches to ongoing injury are different from those for static, residual damage. For this reason, there is a potential role for more objective approaches to evaluating ongoing CNS disease, including CSF biomarkers and functional brain imaging that provide complementary approaches to this problem [13]. While several CSF biomarkers of neural injury have been studied in HIV-infected patients, perhaps the most promising is the light subunit of the neurofilament protein (NFL) [14], [15]. This major structural component of myelinated axons is essential to maintain axonal caliber and to facilitate effective nerve conduction [16], and its concentration in CSF provides a sensitive marker of CNS injury in a number of neurological diseases [17]–[19]. Earlier studies have reported substantially increased levels of CSF NFL in patients with HAD, and mild elevations in some untreated, neurologically asymptomatic patients, mainly in those with low CD4 cell counts, indicating subclinical brain injury [14]. Elevated CSF NFL has been found in some patients up to two years before onset of HAD and thus has the potential to serve as a predictive biomarker of symptomatic progression [15]. Finally, normalization of CSF NFL has been shown both in patients with and without HAD after initiation of cART, [20].

Recently, a new and more sensitive immunoassay for measuring CSF NFL has been introduced. This assay is more sensitive, and in particular, has a broader dynamic range at the lower level of biological concentrations, CSF NFL can now be measured in most normal subjects allowing the detection of smaller increases in pathological states. In this study we applied this new assay to examine the frequency of neuronal injury at different stages of untreated systemic HIV infection and the effects of antiretroviral treatment. Because of the natural increase of CSF NFL concentration with age in the uninfected population, we also examined the relation of HIV-associated NFL changes to subject age. We further examined whether CSF NFL was associated with the level of systemic and CNS viral replication as measured by plasma and CSF HIV RNA concentration, with systemic immunosuppression as reflected in blood CD4+ T cell counts, with intrathecal immune activation measured by CSF neopterin concentrations[21] and blood-brain barrier (BBB) dysfunction assessed by the CSF:blood albumin ratio [22].

## Methods

### Study design and subjects

The report describes two related studies, one cross-sectional and the other a longitudinal cohort design. Both used archived specimens and data from volunteer subjects from two academic centers: Sahlgrenska University Hospital in Gothenburg, Sweden and San Francisco General Hospital, California, USA. All subjects were studied under research protocols approved by institutional review boards of the Sahlgrenska Academy and University of California Committee on Human Research and followed guidelines of the Helsinki declaration. Informed verbal consent was obtained from all patients and documented in their respective patient file, a procedure accepted by the ethic committees. Written consent was not required by the ethic committees. All patients received written and verbal study information.

In the cross-sectional study, 252 HIV-infected subjects were segregated into 6 subgroups (Table 1): Four groups of untreated patients without overt neurological disease (henceforth referred to as neuroasymptomatic, or *NA*) subdivided according to levels of blood CD4+ T-cells into <50 CD4+ T-cells per μL (n = 42), 50–199 cells per μL (n = 49), 200–349 cells per μL (n = 52) and >350 (n = 57) cells per μL; a group of HAD patients (n = 14); one group on suppressive cART with plasma HIV-RNA <50 copies/mL for at least one year (n = 85). Forty-six subjects were included in both untreated and treated groups sampled at different time points, usually several years apart (median 5.9 years, IQR 2.6–9.2). We also included healthy HIV-negative controls (n = 204), table 1. Lumbar puncture was done as part of the clinical follow-ups, at least annualy. Patients were defined as *neuroasymptomatic* if they were without symptoms or signs of neurological or cognitive disability on clinical examination and follow-up. Neuropsychological examinations were performed only on subjects with symptoms or complaints consistent with neurocognitive disorder. The diagnosis of HAD was based on CDC and American Academy of Neurology Task Force Criteria using standard laboratory and clinical evaluations [9], [23], [24].

**Table 1:** Background subject characteristics.

Groups	N	Age	Plasma HIV RNA	CSF HIV RNA	Blood CD4+ T cells
		*Median years (IQR)*	Median Log10 (IQR)		Median cells/μL (IQR)
**Cross-sectional study**					
NA, CD4<50	42	39 (36–49)	5.37 (4.88–5.61)	3.12 (2.30–3.81)	23 (10–40)
NA, CD4 50–199	49	39 (34–48)	4.92 (4.49–5.52)	4.92 (4.49–5.52)	130 (97–164)
NA, CD4 200–349	52	40 (33–50)	4.88 (4.30–5.35)	4.88 (4.30–5.35)	252 (230–300)
NA, CD4 >350	57	42 (34–48)	4.30 (3.74–4.85)	3.43 (2.92–3.92)	490 (400–630)
HAD	14	47 (38–53)	5.02 (4.73–5.38)	5.02 (4.73–5.38)	127 (35–160)
Treated-suppressed	85	47 (38–53)	1.30 (1.30–1.30)	1.30 (1.30–1.30)	530 (330–708)
HIV negative	204	36 (28–52)	NA	NA	NA
**Longitudinal cohort study**					
All subjects	78	40 (34–47)	5.16 (3.98–5.57)	3.91 (3.25–4.44)	190 (70–270)
Normal CSF NFL	52	40 (34–46)	4.95 (4.33–5.39)	4.07 (3.41–4.60)	210 (125–275)
Elevated CSF NFL	26	42 (36–49)	5.43 (5.02–5.88)	3.71 (2.73–4.39)	150 (2

NA: Not available.

The longitudinal cohort study included 78 neuroasymptomatic patients who were treatment-naive or had been off treatment for >6 months. CSF samples were analyzed before and after treatment initiation, with a median time of 15 weeks (interquartile range, IQR 14–23). Subject characteristics are also shown in Table 1.

### Analytical methods

CSF samples were submitted to low-speed centrifugation to remove cells, aliquoted, and frozen to −70°C within 1 hour of performing the lumbar puncture for storage until analysis. CSF NFL protein was measured in the Laboratory of Neurochemistry at the University of Gothenburg using a novel, sensitive sandwich ELISA method (NF-light® ELISA kit) as described by the manufacturer (UmanDiagnostics AB, Umeå, Sweden). The lower limit of quantification was 50 ng/L and intra- and inter-assay coefficients of variation were 4·1–13%. The upper normal refence limits of CSF NFL were <380 ng/L (18–30 years), <560 (30–39 years), <890 (40–59 years), <1850 (>59 years); these limits were established using 108 CSF samples from neurologically healthy control individuals aged 18–76 years.

HIV RNA in CSF and plasma was measured using the Roche Amplicor Monitor version 1.5, Roche Taqman assay version 1 or 2 (Hoffman La-Roche, Basel, Switzerland) or Abbott RealTi*m*e HIV-1 assay (Abbott Laboratories. Abbott Park, Illinois, U.S.A.). The lowest limit of detection was defined as 20 copies per mL. Neopterin was analyzed in CSF using a commercially available immunoassay (BRAHMS, Berlin, Germany) with an upper normal reference value of 5.8 nmol/L [21]. Other measurements, including blood CD4+ T-cell counts, CSF WBC and CSF and blood albumin values to calculate the CSF:blood ratio, were performed in the local clinical laboratories.

### Statistical analysis

The Mann-Whitney test was used to compare variables between independent groups. Wilcoxon matched-pairs signed rank test was used to compare CSF NFL levels before and after treatment. The relationship between log_10_ CSF NFL levels and CD4+ cell counts were analyzed with non-linear regression. The relationship between log_10_ CSF NFL levels and age in controls and the partly overlapping treated and untreated groups were analyzed with a linear mixed effects model.

Predictors of log_10_ CSF NFL were analyzed by multiple linear regression analysis with backward elimination in treated and untreated groups separately. In these analyses WBC and HIV RNA were log transformed and CD4+ T-cell count was inverse transformed (1/(CD4 + 45.3)) based on the previous non-linear regression. General descriptive statistics are presented as median and interquartile range (IQR). All statistical analyses were performed using IBM SPSS Statistics© version 20 or Prism© version 5 (Graphpad Software Inc, La Jolla, CA).

## Results

### Cross sectional study

**Figure 1:**
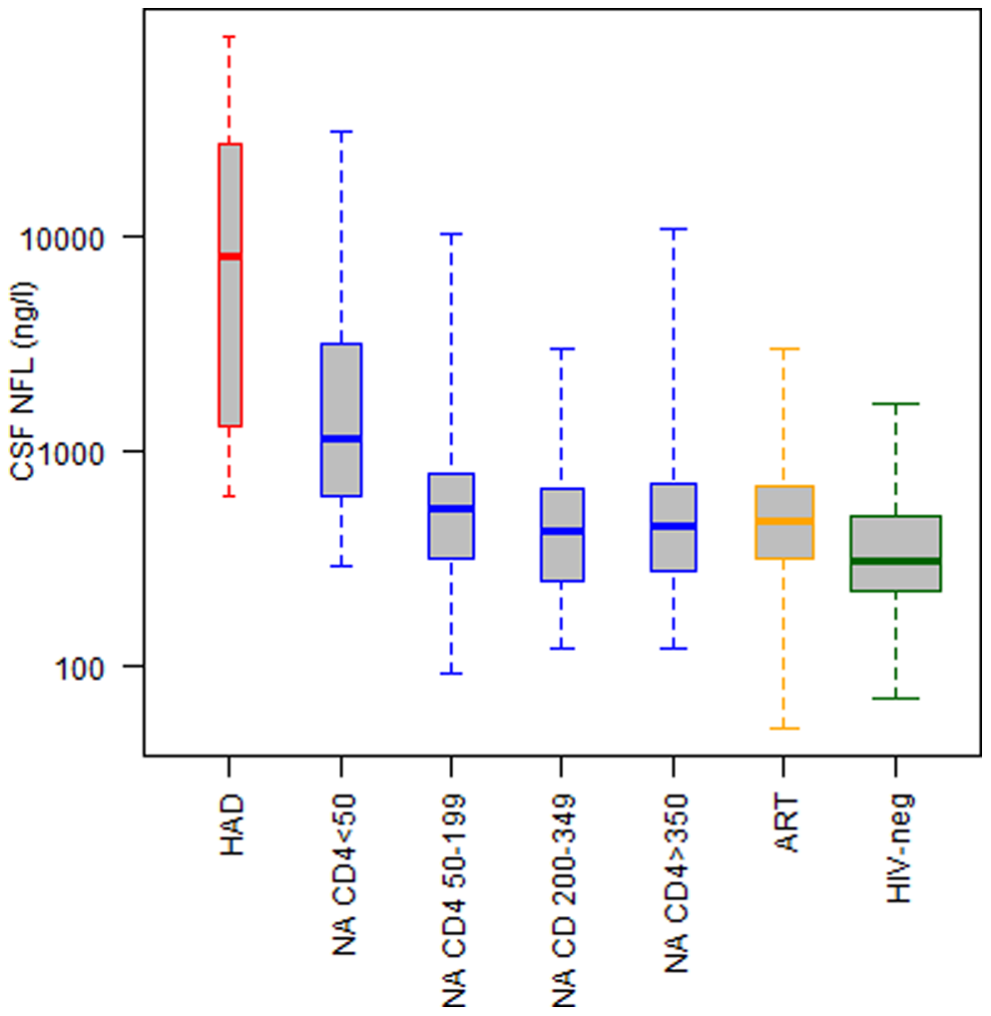
Cross-sectional analysis of CSF NFL in HIV disease. Included were 14 subjects diagnosed with HIV-associated dementia (HAD); HIV positive neuroasymptomatic subjects (NA) without antiretroviral treatment stratified according to levels of blood CD4 T-cells <50 (n = 42), 50–199 (n = 49), 200–349 (n = 52) and >350 (n = 57); 85 subjects on combination antiretroviral treatment (ART) for at least one year and plasma HIV-RNA <50 copies/ml and 204 HIV seronegative volunteers (HIV-neg). CSF NFL concentrations were higher in patients with HAD compared to all other groups. Elevated levels of CSF NFL was also found in subjects with a CD4+ T-cell count below 50 cells/mL compared to groups in higher CD4+ T-cell count strata. Whiskers represent full range

252 HIV-positive patiens subdivided into three groups were studied: Neuroasymptomatics, further stratified into four groups according to levels of blood CD4 cells, HAD and patients on suppressive antiretroviral treatment. 204 HIV-negative subjects served as controls. For further subject characteristics, see table 1. CSF NFL concentrations were higher in subjects with HAD than all other groups including the untreated subjects with equivalently low CD4+ T-cell counts (p<0.001). Higher levels of CSF NFL were also found in subjects with a CD4+ T-cell count below 50 cells/mL compared to the three untreated groups with higher CD4+ T-cell counts (p<0.001) (Figure 1).

Using the laboratory age-related cutoffs for CSF NFL described earlier, nearly all (13/14, 93%) of the HAD patients had elevated CSF NFL concentrations. Additionally, elevated CSF NFL concentrations were found in a substantial number of the untreated neuroasymptomatic subjects, particularly those with lower blood CD4+ T cell counts. Thus, 29/42 (69%) of those with CD4+ T-cell count below 50 cells/mL and 15/49 (31%) with 50–199 CD4+ cells/mL had elevated concentrations, and even 11/52 (21)% of subjects with 200–349 CD4 cells and 11/57 (19%) of those with CD4 cell counts above 350 cells/mL were also elevated—indicating a high level of clinically unappreciated ongoing CNS injury that varied with degree of systemic disease progression. By comparison, in the treated group and HIV-negative subjects the frequency was much lower, 7/85 (8%) and 4/204 (2%), respectively.

#### Correlations of CSF NFL with CD4+ T-cell counts

**Figure 2:**
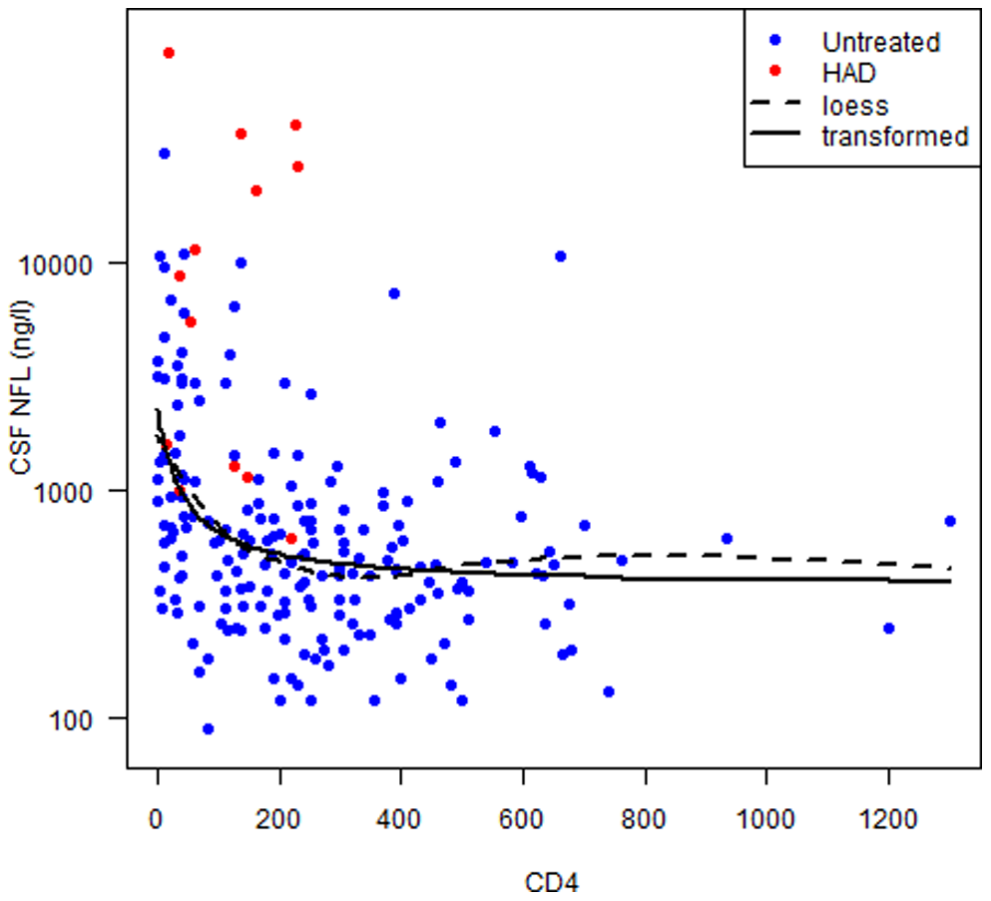
Correlations with CD4 T-cell counts and CSF NFL. A loess regression (dotted line) suggested an inverse transformation of CD4+ T-cell counts. Visual inspection reveals a rapid decline with increasing CD4+ T-cell counts that flattens out at around 250 cells/mL. The relationship between log_10_ CSF NFL levels and CD4+ cell counts were fitted with non-linear regression using the function Log CSF NFL  =  b1 + b2/(CD4 + b3) (filled line). CSF NFL concentrations in patients with HIV-associated dementia (HAD) diagnosis, marked with red color in the figure were significantly elevated also compared to untreated neuroasymptomatic patients with equivalently low CD4+ T-cell counts, p<0.001.

The influence of disease progression was more directly shown as a strong correlation of CSF NFL with the blood CD4+ T-cell counts in neuroasymptomatic untreated patients, p = <0.001. A loess regression suggested an inverse transformation of CD4+ T-cell counts (Figure 2). Visual inspection revealed a rapid decline with increasing CD4+ T-cell counts that flattens out at around 250 cells/mL. CSF NFL concentrations in patients with HIV-associated dementia (HAD) diagnosis, marked with red color in figure 2, were significantly higher also compared to neuroasymptomatic patients with equivalently low CD4+ T-cell counts, p<0.001, suggesting additional factors contributing to this more severe and clinically overt disease.

No significant correlation was found between CD4+ T-cell counts and CSF NFL within the treated, virologically suppressed group.

#### Correlations of CSF NFL with age and treatment effects

**Figure 3:**
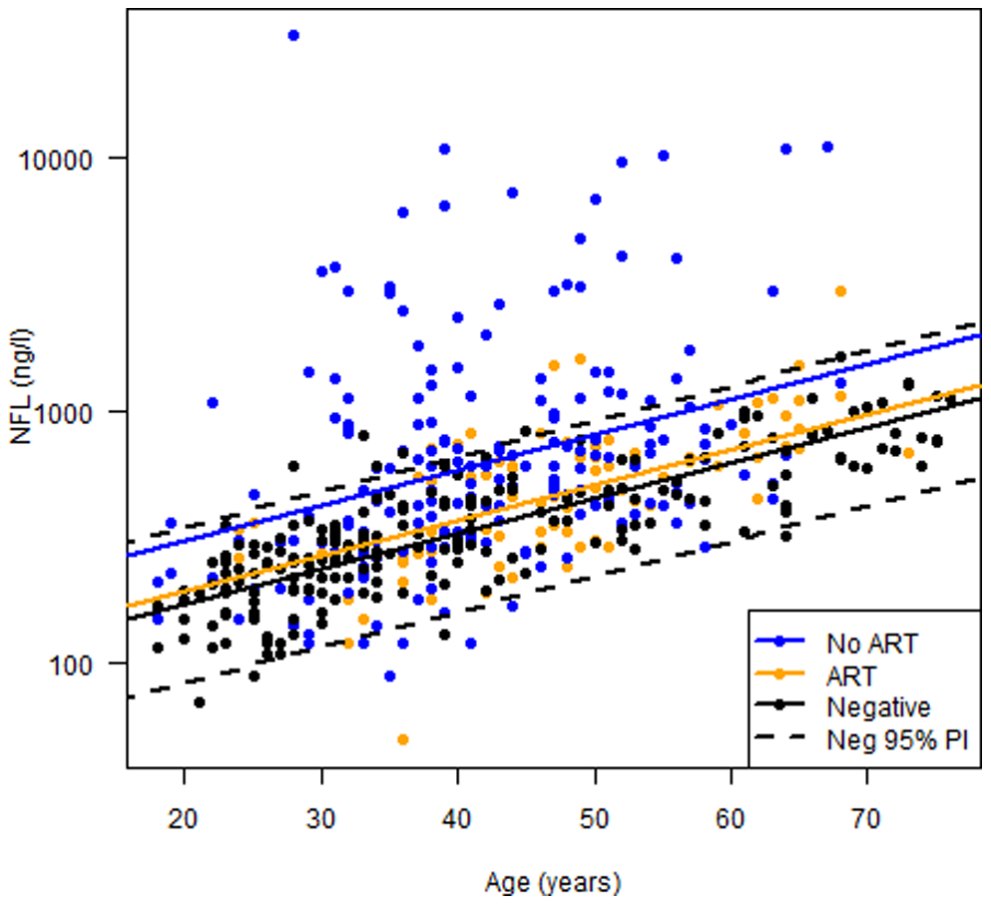
CSF NFL related to age and treatment effect. Since CSF NFL increases with age, we analyzed the group differences with a linear mixed effects model with age as covariate. This implies a model with three parallel regression lines where the group differences correspond to the vertical distances between the regression lines. The group differences can be expressed as the corresponding age increase needed for an equivalent difference. The 95% prediction interval of CSF NFL levels of HIV-negative controls is demonstrated as dotted lines (Neg 95% PI). Concentrations of CSF NFL in neuroasymptomatic untreated HIV-infected subjects (No ART) were equivalent to those of HIV-negative subjects (negative) who were 18.5 years older (p<0.001). CSF NFL concentrations in the treated group (ART) were equivalent to those of HIV-negative subjects who were 3.9 years older (p<0.01).

Since CSF NFL increases with age in uninfected populations, we analyzed the differences between treated and untreated subjects and HIV negatives with age as covariate. This resulted in a model of three parallel regression lines (Figure 3). The group differences can be expressed as the corresponding age increase needed for an equivalent difference in the two HIV-infected groups compared to the controls.. We found that the CSF concentration of NFL in the untreated group, despite their lack of symptoms, was equivalent to subjects 18.5 years older than the HIV negative controls (p<0.001). While treatment reduced these concentrations, CSF NFL concentration in the population on cART was equivalent to HIV-negatives 3.9 years older (p<0.01), figure 3. The 95% prediction interval for CSF NFL levels in HIV-negative controls corresponded well to the age-related cut-offs provided by the laboratory.

#### Multivariate analysis

A multiple non-linear regression analysis that included CSF NFL versus age, CD4+ T-cell counts, CSF WBC, CSF:blood albumin ratio, plasma and CSF HIV RNA and CSF neopterin was performed on both untreated and treated patients. Univariate and multivariate analyses are shown in Table 2. In the treated group CD4+ T-cell nadir and pre-treatment HIV RNA in CSF and plasma were also included in the analysis. In the untreated asymptomatic groups, age (n = 200), CD4+ T-cell counts (n = 200), CSF neopterin (n = 188), CSF WBC count (n = 200) and CSF/plasma albumin ratio (n = 200) all stood out as possible independent predictors related to CSF NFL in the univariate analysis. These predictors were also statistically significant with adjusted estimates in the multivariate analysis. By contrast the plasma and CSF viral loads were not significant predictors. In the treated group, only age (n = 85), pre cART plasma viral load (n = 63) and CSF/plasma albumin ratio (n = 85) were found as independent predictors of CSF NFL concentration (Table 2).

**Table 2:** Univariate correlation (left columns) and multiple linear regression (right columns) determining predictors of log_10_ CSF NFL in HIV-infected neuroasymptomatic patients with and without antiretroviral treatment.

		Univariate		Multivariate	
Variable	N	Std β (r)	P	Std β_adj_	P_adj_
**No treatment**					
Age	200	0.35	<0.001	0.26	<0.001
Blood CD4*	200	0.43	<0.001	0.36	<0.001
CSF neopterin	188	0.31	<0.001	0.23	<0.001
Log CSF WBC	200	−0.27	<0.001	−0.17	0.009
Albumin ratio	200	0.33	<0.001	0.33	<0.001
Log plasma VL	198	0.29	<0.001	NS	NS
Log CSF VL	199	−0.036	0.62	NS	NS
**Treated-suppressed**					
Age	85	0.66	<0.001	0.57	<0.001
Blood CD4*	85	0.089	0.42	NS	NS
CSF neopterin	77	0.062	0.59	NS	NS
Log CSF WBC	85	−0.051	0.65	NS	NS
Albumin ratio	85	0.4	<0.001	0.26	0.004
Log pre ART plasma VL	63	0.16	0.23	0.23	0.013
Log pre ART CSF VL	46	−0.31	0.23	NS	NS
Log CSF VL	85	−0.091	0.41	NS	NS
Nadir CD4*	61	0.023	0.023	NS	NS

*CD4+ T-cell count was inverse transformed (1/(CD4+45·3) NS = Not significant.

### Longitudinal Cohort study

**Figure 4:**
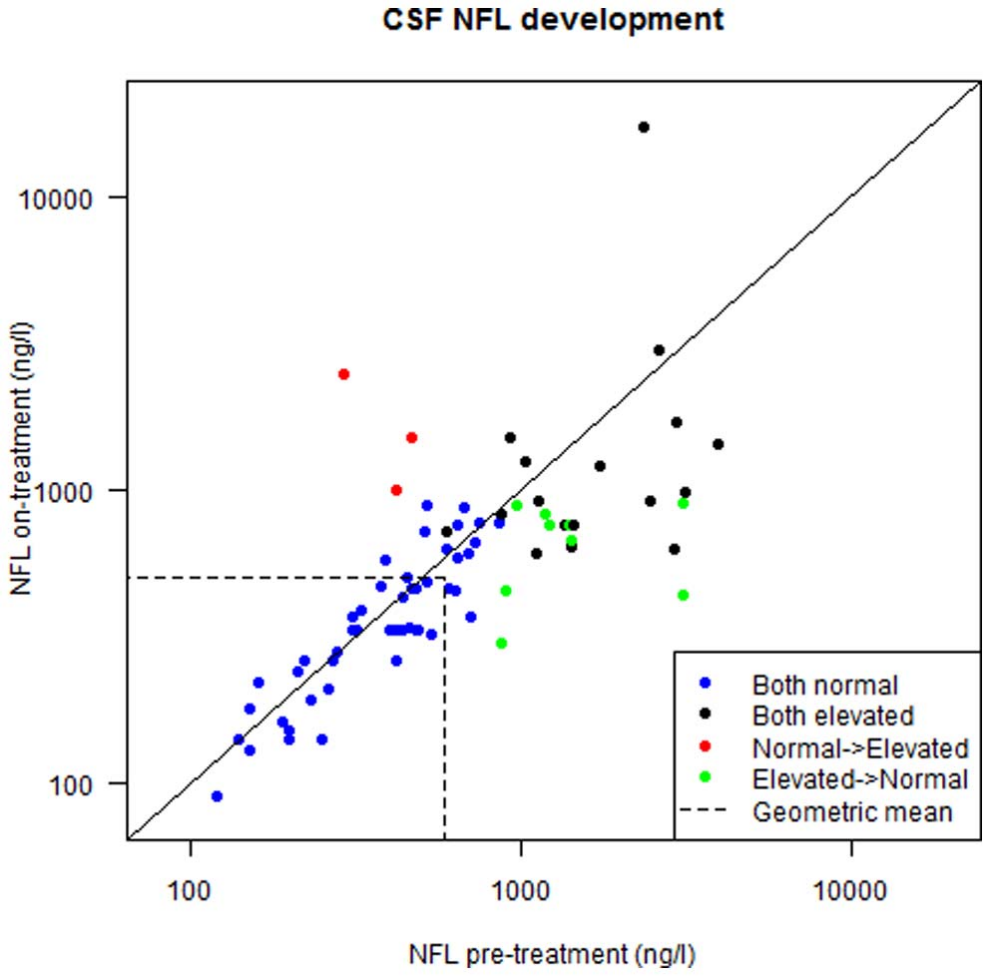
CSF NFL levels before-after combination antiretroviral treatment (cART) initiation. Overall CSF NFL levels decreased in 63% of the patients after initiation of cART (p<0.01), demonstrated as a lower CSF NFL geometric mean (dotted line) after initiation of cART. 33% of the patents had elevated levels of CSF NFL at baseline and 81% of those exhibited reduction in their CSF NFL levels after treatment (p<0.01). 35% of patients with pathological CSF NFL at baseline normalized their levels (green colored). Those with normal CSF NFL at baseline exhibited no significant reduction in CSF NFL (blue colored). Three patients with normal baseline CSF NFL exhibited elevated levels of CSF NFL after cART initiation (red colored).

Overall, CSF NFL levels decreased in 63% of the patients (n = 49) from a pre-treatment CSF NFL median level of 520 ng/L (IQR 310–1070) to 475 ng/L (IQR 315–782.5) after initiation of cART (p<0.01) (Figure 4). Of the 26 (33%) subjects with elevated levels of CSF NFL at baseline, 21 (81%) exhibited a reduction in their CSF NFL levels after treatment, from a pre-treatment median level of 1415 ng/L (IQR 1023–2715) to a posttreatment level of 825 ng/L (IQR 662.5–1220) after a median of 15 weeks of treatment (p<0.01), figure 4. Nine (35%) had normalized by the posttreatment assessment. In one patient, an unexpected increase from 2350 ng/L to 17310 ng/L was observed. This patient had systemic Mycobacterium Avium Complex infection and developed systemic immune reconstitution inflammatory syndrome (IRIS) after starting cART which may have also involved the CNS.

Although CSF NFL decreased in some patients with normal baseline CSF NFL (n = 52), overall there was no significant reduction for the group as a whole. All patients with normal CSF NFL concentrations at baseline remained normal except for three subjects who exhibited increases to abnormal levels from 470 to 1500 ng/L, from 290 to 2460 ng/L and from 420 to 990 ng/L, respectively (reference range for their ages is <890 ng/L). These changes were not accompanied by notable neurological symptoms and all three showed decrease in plasma and CSF HIV RNA and exhibited increases in their CD4 counts after treatment. The patient with the highest CSF NFL increase developed a cutaneous vasculitis, along with a proximal venous thrombosis requiring anticoagulants and high doses of corticosteroids. No symptoms and signs associated with immune reconstitution or other disorders to explain the elevations were noted. One patient was lost to follow-up.

## Discussion

Our results provide compelling evidence supporting the value of CSF NFL as a biomarker of axonal disruption in untreated patients with HIV infection. They show that CSF NFL is not only a sensitive biomarker of symptomatic HAD, but also demonstrates that a substantial number of untreated asymptomatic patients suffer subclinical CNS injury. The prevalence of this injury increases as systemic disease progresses and blood CD4+ T cells fall. Thus, CSF NFL was strongly inversely correlated with CD4+ T-cell counts. Indeed, a clear majority of patients with counts below 50 cells/μL exhibited elevation of this neuronal marker in CSF and nearly half of those with counts below 200 had abnormal levels showing that subclinical injury in more advanced disease is common.

This common, presumably chronic, brain injury at reduced cell counts provides one explanation for the reported high prevalence of neurological symptoms and reduced cognitive performance in treated patients with lower CD4+ T cell nadirs [25]–[27] as it leaves behind a legacy of irreversible damage prior to treatment initiation underlying subsequent function. The fact that we did not find a correlation between CSF NFL and CD4+ T-cell nadir, itself, is consistent with this earlier injury rather than the nadir count predisposing to subsequent ongoing pathology. Moreover, the substantial proportion of patients with even higher CD4+ T-cell counts (>200 cells/μL) with elevated CSF NFL levels may imply a less common and less severe, but still significant, neuropathological insult even in subjects with relatively preserved immunity. Although the prognostic meaning of these generally milder elevated CSF NFL levels in asymptomatic HIV infected patients was not studied here, a previous study showed that increased CSF NFL was found to be predictive of HAD development in neuroasymptomatic patients compared to CD4-matched controls in a case-control study [15]. It is therefore likely that asymptomatic patients with increased CSF NFL suffer subclinical HIV-related neurodegeneration that results in cumulative injury and later disability in some patients. While treatment of those with elevated NFL may reverse this, it may leave a legacy of compromised brain integrity and function. These results therefore provide strong, albeit uncontrolled, support for early treatment initiation to prevent subsequent neurological dysfunction.

CSF NFL increases with age in the absence of HIV infection. Though the mechanisms underlying this increase are not established, it may confound analysis of the consequences of the HIV infection itself and potential treatment effects on CSF NFL over time. However, when comparing neuroasymptomatic untreated patients as a whole to HIV-negative controls and the HIV-positive group on cART, CSF NFL was higher in untreated subjects regardless of age, equivalent to 18.5 years older HIV negative controls. Fortunately, this elevation is largely reversible. Yet, and perhaps of greater interest, levels of CSF NFL in the treated and virologically suppressed group remained significantly higher than those of the HIV-negative population, a difference equivalent to the addition of 3.9 years of life in this group. This finding might reflect a discrete, continuous neuroinflammation and axonal degradation due to ongoing, yet undetected, infection or continued local infection with mild, but important neuropathogenic effect. Or it might indicate an effect of the prior injury that is similar to the effect of aging on the metabolism of NFL and their neuronal substrate. Apart from neuroinflammatory and neurodegenerative diseases, such as MS [28], [29] and frontotemporal dementia[28], [29], NFL is also increased in acute damage to the CNS, for example following diffuse axonal injury or stroke [30], [31]. The increase is generally interpreted as reflecting a direct, destructive effect on NFL-rich large caliber axons. Although this is likely in our study as well, we cannot exclude that the noticed increase of NFL in part depends on other factors, such as reparative processes, in which injured axons are slowly removed leading to a prolonged release of NFL into the CSF. Since NFL was analyzed in CSF, it reflects CNS concentrations, although a minimal part might derive from peripheral nerve roots in connection with the CNS.

Future studies of both aging and treated HIV infection need to address the sources of this elevated CSF NFL, whether due to increased release or turnover of NFL or, alternatively, reduced clearance from CSF. Additionally, it will be important to assess correlations of elevated CSF NFL in treated patients with detailed neuropsychological test performance in addition to pretreatment neurological status to dissect the effects of earlier injury from evidence of ongoing damage in determining post-treatment neurological status and CSF NFL.

Earlier studies have shown that microglia/macrophage activation, reflected by increased levels of CSF neopterin is an important factor in HIV neuropathogenesis [21]. In untreated patients, CSF neopterin concentrations are almost always elevated and increase progressively as immunosuppression worsens and blood CD4+ T-cell counts drops [21]. In our data, CSF neopterin and CSF WBC count were strongly and independently correlated with CSF NFL in untreated patients. This suggests that the intrathecal inflammatory reaction is important for the development of axonal degradation in HIV infection and support the neuropathogenic role of macrophage/microglial activation.

CSF neopterin was not found to be a predictive factor for CSF NFL in treated patients. However, CSF albumin ratio was an independent predictor for CSF NFL in both treated and untreated HIV infected subjects. Increased CSF albumin ratio reflects a compromised BBB with increased permeability of serum proteins and other substances into the brain. Perivascular inflammation and BBB dysfunction are frequent findings in patients with HAD [30]–[32] and our data suggest that increased permeability across the BBB may be pathogenically linked to axonal injury in HIV.

Several reports indicate that milder forms of HAND are fairly common also in HIV infected patients on effective treatment [11], [33], [10]. These reports are however based solely on neuropsychological testing. Data from this study give more objective indications that also virologically suppressed patients are at risk of CNS damage. However, confounders that we are not aware of could be present and it is important to point out that only a small fraction of treated subjects had pathological levels of CSF NFL.

There are a number of limitations of this study. It involved a convenience sample studied through archived samples. Importantly, extensive neuropsychological testing required for Frascati criteria diagnosis [9] was not performed. The cross-sectional study also has its limitations in assessing treatment effects, though the longitudinal cohort study shows that NFL decreases after treatment initiation, thereby clearly linking NFL elevations with CNS HIV infection.

## Conclusions

The findings of this study support the value of CSF NFL as an independent marker of CNS damage at different stages of HIV infection. We present data indicating subclinical brain injury reflected by levels of CSF NFL in a substantial proportion of untreated HIV infected patients. The most important influencing factors in addition to subject age were CD4+ T-cell counts, CSF neopterin levels, CSF WBC counts and CSF/plasma albumin ratio, reflecting the importance of systemic disease progression, immune activation and blood brain barrier dysfunction in HIV neuropathogenesis. Antiretroviral treatment has a substantial effect on intrathecal immunoactivation and neurodegeneration, but even virologically suppressed patients exhibited higher levels of CSF NFL than HIV negative controls, suggesting ongoing low-grade injury or an aging effect of HIV infection that is not fully reversed by treatment. To better understand the prognostic meaning of fluctuations of CSF NFL in HIV disease and to evaluate this marker as a diagnostic tool for HIV-associated neurocognitive disorders, future prospective studies with concurrent neuropsychological testing will be important.
